# mHealth Apps for Self-Management of Cardiovascular Diseases: A Scoping Review

**DOI:** 10.3390/healthcare10020322

**Published:** 2022-02-08

**Authors:** Nancy Aracely Cruz-Ramos, Giner Alor-Hernández, Luis Omar Colombo-Mendoza, José Luis Sánchez-Cervantes, Lisbeth Rodríguez-Mazahua, Luis Rolando Guarneros-Nolasco

**Affiliations:** 1 Tecnológico Nacional de México/I. T. Orizaba, Av. Oriente 9, No. 852, Col. Emiliano Zapata, Orizaba 94320, Mexico; dci.ncruz@ito-depi.edu.mx (N.A.C.-R.); lrodriguezm@ito-depi.edu.mx (L.R.-M.); luisguarneros@gmail.com (L.R.G.-N.); 2 Tecnológico Nacional de México/Instituto Tecnológico Superior de Teziutlán, Fracción l y ll, Teziutlán 73960, Mexico; luis.cm@teziutlan.tecnm.mx; 3 CONACYT-Tecnológico Nacional de México/I. T. Orizaba, Av. Oriente 9, No. 852, Col. Emiliano Zapata, Orizaba 94320, Mexico; jlsanchez@conacyt.mx

**Keywords:** cardiovascular diseases, mHealth, self-management

## Abstract

The use of mHealth apps for the self-management of cardiovascular diseases (CVDs) is an increasing trend in patient-centered care. In this research, we conduct a scoping review of mHealth apps for CVD self-management within the period 2014 to 2021. Our review revolves around six main aspects of the current status of mHealth apps for CVD self-management: main CVDs managed, main app functionalities, disease stages managed, common approaches used for data extraction, analysis, management, common wearables used for CVD detection, monitoring and/or identification, and major challenges to overcome and future work remarks. Our review is based on Arksey and O’Malley’s methodological framework for conducting studies. Similarly, we adopted the PRISMA model for reporting systematic reviews and meta-analyses. Of the 442 works initially retrieved, the review comprised 38 primary studies. According to our results, the most common CVDs include arrhythmia (34%), heart failure (32%), and coronary heart disease (18%). Additionally, we found that the majority mHealth apps for CVD self-management can provide medical recommendations, medical appointments, reminders, and notifications for CVD monitoring. Main challenges in the use of mHealth apps for CVD self-management include overcoming patient reluctance to use the technology and achieving the interoperability of mHealth applications with other systems.

## 1. Introduction

According to the World Health Organization (WHO), cardiovascular diseases (CVDs) are a group of disorders of the heart and blood vessels. They affect the normal behavior of the organism and have an adverse impact on a patient’s emotional wellbeing, as well as on their work, family, social, and economic environments. On a much larger scale, CVDs are a public health concern due to their high prevalence, mortality, vulnerability, and the high public costs implied in their management. According to the WHO, CVDs are and will remain the number one cause of death globally at least for the following eight years. In fact, by 2030 almost 23.6 million people are estimated to die from some form of CVD [1]. In 2017 alone, approximately 17.9 million people died from CVDs, representing 31% of all global deaths. Overall, 85% of CVD-related deaths are due to either heart attacks or strokes. People suffering from CVDs or those at greater risk of developing them need to rely on effective means, such as counseling and medicines, for early CVD detection and management.

Mobile health (mHealth) is a medical and public health practice supported by mobile devices, such as mobile phones, portable monitoring devices, and personal digital assistants. It involves using strategies such as smartphone apps, global positioning systems (GPS), and Bluetooth technologies. Approximately 500 million patients use mobile health (mHealth) applications to support their self-healthcare activities [2]. In this sense, cardiovascular mHealth is the most used in the mHealth domain through innovation, research, and implementation in the areas of CVD prevention, cardiac rehabilitation, and education [3]. Additionally, the most promising domains of mHealth use have to do with blood pressure monitoring, cardiac rehabilitation, arrhythmia monitoring, medication management, and social support.

mHealth apps hold promise for delivering health information and services to patients, especially for chronic diseases such as CVDs, which require extensive self-management. Self-management is key to person-centered care, but its support requires an understanding of individual preferences for different types of health information and decision-making autonomy. The self-management of chronic conditions requires the ability to manage the symptoms, treatment, physical, and psychosocial consequences and lifestyle changes inherent to living with a chronic condition. Additionally, self-management is inherent to person-centered care that promotes a balanced consideration of the values, needs, expectations, preferences, capacities, health, and wellbeing of all the constituents and stakeholders of the healthcare system. Effective self-management and person-centered care require full accommodation of people’s needs and preferences for different types and amounts of information and other care services, a degree of autonomy in health-related decision-making, and support from their healthcare professionals and family members [4].

Current studies investigating mHealth interventions for patients with CVDs have returned mixed findings. Hence, more effort and work are needed to create engaging mHealth platforms that provide the necessary level of support to make sustained behavioral change. Similarly, addressing specific motivational, physical, and cognitive barriers to mHealth adoption among patients might increase the utilization of future interventions. It is also important to adopt new approaches that minimize the weaknesses of commercially available mobile apps [5].

We found related reviews that are focused on mobile apps for CVDs self-management using different technologies. These reviews studied the impact of incorporating mobile applications for symptom tracking, medication reminding, self-care support, and physiological state monitoring on the self-management of CVD patients’ health. In addition, we identified that most of these proposals addressed the prevention and treatment of heart failure, arrhythmias, and coronary disease. Searcy et al. [5] documented the use domains of mHealth in CVD management, the barriers to mHealth adoption in older adults, and future directions for mHealth to increase engagement in this population. Furthermore, other studies [6,7,8,9,10,11] have explored the effectiveness, acceptability, and usefulness of mobile applications for CVD self-management and risk factor control using a variety of performance metrics. These studies have identified the most attractive features of the applications, such as the monitoring of healthy behaviors and the personalization of content. In addition, they have concluded that cardiovascular disease risk factors and behaviors are modifiable in the short term. Other authors [3,4,12] studied the mHealth apps for CVD prevention and management. Likewise, Cruz-Martínez et al. [13] identified interventions of self-management through the use of remote monitoring technologies. Other studies have rather focused on the self-management of specific CVDs. For instance, Refs. [14,15] analyzed the effect of the use of wearables and apps for cardiac rehabilitation of arrhythmia patients, whereas [16,17,18] studied a series of prevention and treatment programs for heart failure management through mHealth. Additionally, in [2,19,20,21,22] the functionalities of mHealth apps for heart failure self-management were evaluated.

The main difference between our scoping review and similar state-of-the-art reviews is that ours addresses more CVDs than those more frequently addressed in other reviews. Our scoping review aims at describing the current state of mHealth apps for CVD self-management by analyzing six aspects: (1) main CVDs managed, (2) main app functionalities, (3) common wearables used with these apps, (4) disease stages managed, (5) common approaches used for data extraction, analysis, and management, and (6) challenges and future work remarks. The review comprises a body of scientific literature issued from 2014 to mid-2021. The remainder of this paper is organized as follows: Section 2 introduces the materials and methods used to conduct the review. In Section 3, we present our results with respect to the research questions, whereas in Section 4, we discuss such findings. Finally, our conclusions are summarized in Section 5.

## 2. Materials and Methods

Our review is based on Arksey and O’Malley’s [23] methodological framework for conducting studies as well as on the recommendations of Levac regarding such a framework [24]. Similarly, we adopted the PRISMA model proposed by Moher et al. [25] for reporting systematic reviews and meta-analyses and the PRISMA-ScR model extension. Next, we relied on the work of Tricco et al. [26] to determine how to organize and present the scoping review findings. The scoping review comprises five development phases: (1) identify research questions, (2) identify relevant studies, (3) select relevant studies, (4) chart the data, and (5) collate, summarize, and report findings.

### 2.1. Research Questions

We formulated seven research questions that framed our scoping review, helped us meet our research goals, and guided us throughout the reviewing process.

- RQ1. Which CVDs are most commonly managed by mHealth apps?
- RQ2. Which mHealth apps for CVD self-management are reported in the literature?
- RQ3. What are the main functionalities of mHealth apps for CVD self-management?
- RQ4. What are the major remarks for future work and challenges to be overcome by mHealth apps for CVD self-management?
- RQ5. Which approaches to data extraction, analysis, and management are commonly implemented in mHealth apps for CVD self-management?
- RQ6. Which wearables are commonly used to detect, monitor, and/or identify CVDs?
- RQ7. Which CVD stages are commonly managed by mHealth apps?

### 2.2. Inclusion and Exclusion Criteria

At the first stage of the search strategy, we defined the repositories in which we would search for the primary studies. These repositories included IEEE Xplore Digital Library, PubMed, ScienceDirect (Elsevier), SpringerLink, and Wiley Online Library. According to our preliminary search, these digital libraries hosted a greater amount of related literature when compared to other repositories, such as ACM (Association for Computing Machinery) Digital Library and Web of Science. Additionally, we relied on Google Scholar to expand our search. At the second stage of the search strategy, we performed a keyword search for primary studies issued within the 2014–2021 period. Table 1 lists such keywords, which were used both individually and combined using the conjunctions “and” and “or” to broaden our results.

The following queries were built to search for primary studies in each selected repository.

1. ‘Cardiovascular disease’ AND (‘Self-management’ OR ‘Self-care’ OR ‘Self-monitoring’) AND (‘mHealth’ OR ‘mobile application’ OR ‘smart application’ OR ‘wearable’ OR ‘smartwatch’ OR ‘app’). The analysis of the preliminary results of this query revealed relevant search terms related to different cardiovascular disease types. Query 2 includes these search terms to expand on the relationship identified.
2. (‘Heart disease’ OR ‘Cardiac issues’ OR ‘Heart failure’ OR ‘Arrhythmia’ OR ‘Coronary heart disease’ OR ‘Atrial Fibrillation’ OR ‘Hypertension’ OR ‘Cardiac arrest’ OR ‘Peripheral artery disease’) AND (‘Self-management’ OR ‘Self-care’ OR ‘Self-monitoring’) AND (‘mHealth’ OR ‘mobile application’ OR ‘smart application’ OR ‘wearable’ OR ‘smartwatch’ OR ‘app’).

Finally, we used the PRISMA model as a guide to organize and report our results.

### 2.3. Study Selection and Eligibility

**Figure 1:**
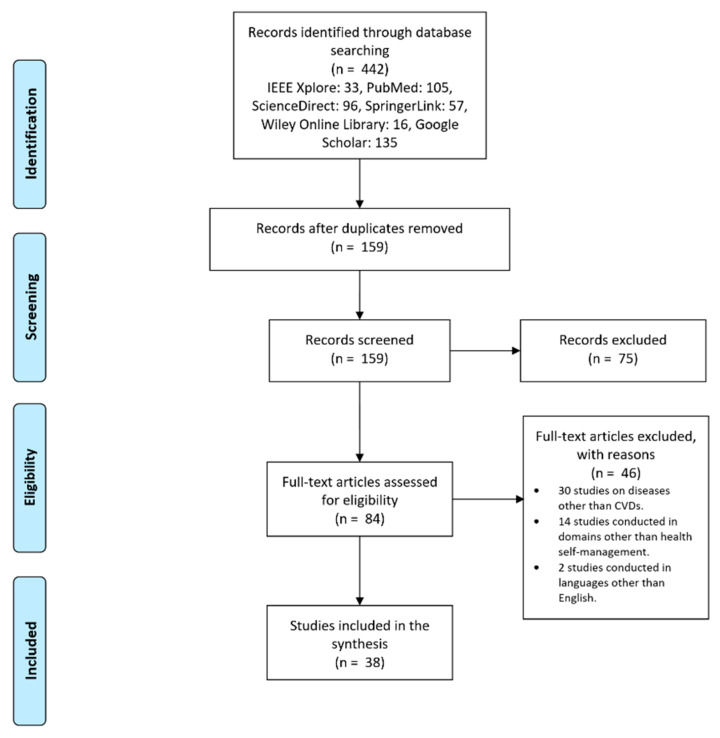
Study selection process—PRISMA diagram flow.

At the end of the search process, we found 442 relevant results: 33 from IEEE Xplore Digital Library, 105 from PubMed, 96 from ScienceDirect (Elsevier), 57 from SpringerLink, 16 from Wiley Online Library, and 135 from Google Scholar. Then, after removing duplicates, we relied on 159 articles for the first analysis, which necessitated classifying these papers by title and abstract. We performed a full-text reading of 84 of these articles, 38 of which were finally used in the scoping review (see Figure 1).

Once we gathered the initial 442 studies, we selected those containing at least two of the keywords listed in Table 1 in their abstract. Then, we removed those papers that were not directly related to CVD self-management. Following this step, we kept just 159 studies: 16 from IEEE Xplore Digital Library, 35 from PubMed, 27 from ScienceDirect (Elsevier), 12 from SpringerLink, 10 from Wiley Online Library, and 59 from Google Scholar. Next, we analyzed these papers with respect to our set of established exclusion criteria:

1. Studies on diseases other than CVDs;
2. Studies conducted in domains other than health self-management;
3. Studies written in languages other than English.

**Figure 2:**
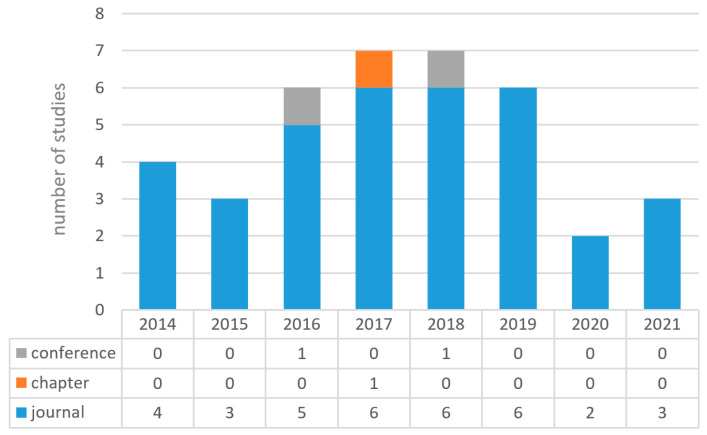
Type of publication from 2014 to 2021.

The remaining 38 primary studies were those comprising the scoping review. We downloaded the entire file of each study to ensure its proper analysis. As depicted in Figure 2, the majority of the studies (92.1%) were published in journals, 2.6% were issued as book chapters, and 5.3% were published in conference proceedings. Moreover, most of the studies were published between 2017 and 2018.

**Figure 3:**
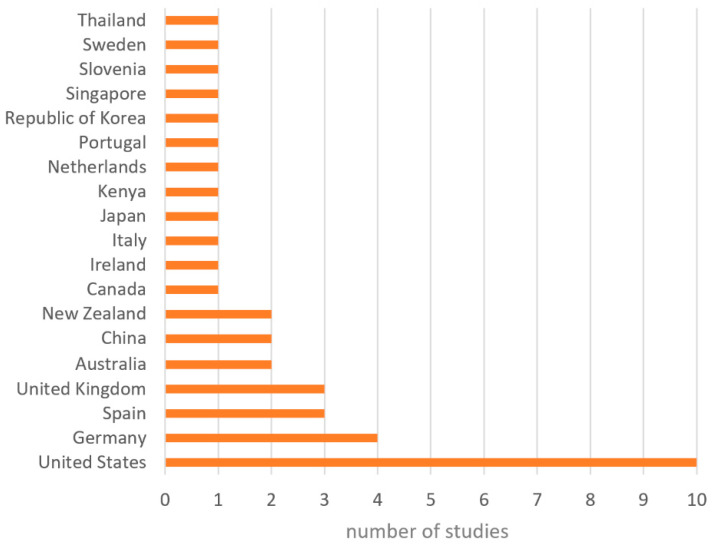
Geographical distribution of primary studies.

Figure 3 illustrates the geographical distribution of our primary studies. As can be observed, most of them were conducted in the United States.

**Figure 4:**
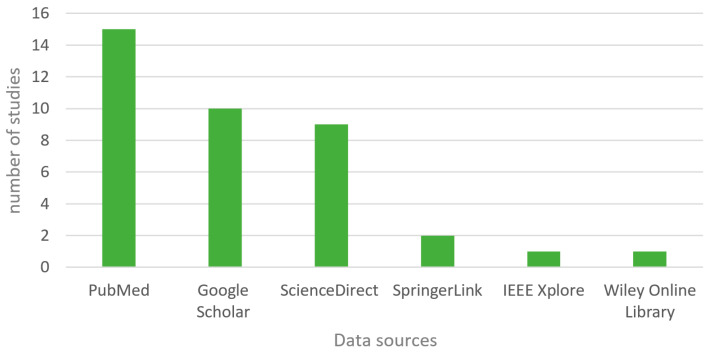
Primary studies by digital libraries.

As regards allocation (see Figure 4), the majority of the primary studies were collected from PubMed, followed by Google Scholar, and then ScienceDirect. Research articles retrieved from SpringerLink, Wiley Online Library, and IEEE were less frequent.

### 2.4. Data Collection and Analysis

Once we defined the primary studies to be used in the review, we retrieved their bibliographic data and content data. The former included research title, authors, research goals, and database of provenance. The latter refer to the information contained in each study helping us answer our research questions (see Section 2.1).

## 3. Results

We reviewed the studies with respect to six aspects (see Table 2) aligned with our research questions. These aspects are listed and briefly explained below:

1. Type of CVD that is managed by each mHealth app.
2. Main app functionalities. Central capabilities of mHealth apps for CVD self-management, including (a) medical recommendations for patient follow-up, (b) real-time alerts before vital sign alterations, (c) medication management, (d) report of monitored parameters, (e) reminders for patient adherence to medication, physical activity, and/or dietary plans, (f) patient–physician communication via text messages, and (g) atrial fibrillation (AF) detection.
3. Challenges and/or future work remarks (when applicable). Main challenges to overcome and/or suggestions for future work for mHealth apps used in CVD self-management.
4. Approaches to data analysis, extraction, and management. The approaches were identified such as (a) machine learning techniques, (b) machine learning tasks, (c) big data types, and (d) device/sensor types. We identified mHealth apps relying on large datasets and big data analysis techniques. Additionally, there are apps relying on machine learning algorithms (MLAs) or techniques. Finally, we detected mHealth apps relying on sensors/wearables to obtain patient data (e.g., vital signs).
5. Device and apps. Information on the wearables and web and mobile apps—either commercially available or purposefully developed in the study itself—used by each mHealth app to retrieve patient data and biomedical variables.
6. CVD phase or set of phases managed by each mHealth app reviewed. The main CVD phases identified were diagnosis, prevention, monitoring, and treatment.

## 4. Discussion

### 4.1. RQ1. Which CVDs Are Most Commonly Managed by mHealth Apps?

The CVDs most commonly managed by mHealth apps in the literature are arrhythmias, heart failure, and coronary heart disease. Arrhythmia self-management is present in 34% of the reviewed studies [39,40,41,42,43,44,45,46,47,48,49,50,51], whereas heart failure self-management exhibited a frequency of 32% [27,28,29,30,31,32,33,34,35,36,37,38]. In turn, coronary heart disease self-management is present in 18% [52,53,54,55,56,57,58]. Additionally, 16% of the papers explore the self-management of several CVDs simultaneously [59,60,61,62,63,64].

We attribute this to the fact that these diseases have a high prevalence and mortality worldwide. In this regard, we believe that researchers are mainly focusing on solutions for the management of arrhythmias, specifically atrial fibrillation, because it affects 25% of the population aged over 40 years.

Some studies analyzed mHealth apps that address more than one cardiovascular disease at a time; we believe that this is due to the patients’ risk factors, which can cause comorbidities, i.e., one disease can develop from another. However, our recommendation is that mHealth applications should focus only on one particular disease to provide more accurate forecasts, as each disease has its own characteristics.

### 4.2. RQ2. Which mHealth Apps for CVD Self-Management Are Reported in the Literature?

As regards arrhythmia self-management, mHealth apps include the RITMIA smartphone app [41], the KB app [43], the mAF app [45], and PULSE-SMART [50]. Generally speaking, these apps issue medical recommendations and allow for the early detection of AF, the most common type of arrhythmia. mHealth apps for arrhythmia self-management generally focus on arrhythmia prevention, diagnosis, and monitoring. The monitoring devices that can be connected to these apps are the T-shirt-type wearable ECG monitor and the AliveCor Kardia Mobile ECG.

mHealth apps for heart failure self-management include the Heart Failure app [27], HeartMan [28], Healthy Heart [29], Evident II [31], Abby [32], MOVIDA.eros [33], HeartKeeper [36], and SUPPORT-HF [37]. The majority of them focus on heart failure monitoring and treatment through issuing medical recommendations and medication management. Oximeters and sensors for blood pressure measurement are the most common devices connected to these apps.

The Care4Heart [53], HeartMapp [54], and Text4Heart [58] apps support coronary heart disease self-management. They primarily issue medical recommendations, reminders, and alerts and offer medication management. Most of these apps focus only on coronary heart disease monitoring. Devices and wearables such as heart rate monitors, the BioHarness Bluetooth sensor, portable ECG monitors, and T-shirts with sensors are usually connected to these applications.

mHealth apps such as those reported in [59,60,61,62,63,64] aim at supporting the self-management of multiple CVDs. These mHealth apps mainly provide medical follow-up recommendations for physical activity or dietary plans. Likewise, they issue medication reminders and real-time warnings before potential vital sign alterations. We found that 63.6% of the mHealth apps are compatible with the Android operating system, whereas 13.6% support iOS, and 22.8% support both (see Table 3).

It is reasonable to believe that there are more mHealth applications for the Android operating system because it is the most popular operating system in the world. However, we found in this research that the most complete mHealth application is the Kardia app, which is available for the iOS operating system only. Therefore, we believe that it is important to develop cross-platform mHealth applications; in this regard, an alternative would be the use of PWA (progressive web app) development technologies.

Another remarkable finding is that only 2 of the 16 mobile applications analyzed are available through digital distribution platforms: (1) MOVIDA.eros and (2) the Kardia app. Moreover, some of the applications analyzed were subjected to user acceptance tests with small groups of patients; in addition, some of them are not widely available because they are still in development. In this regard, we believe there is an opportunity to release free trial versions of the mHealth applications to test them with larger patient samples.

### 4.3. RQ3. What Are the Main Functionalities of mHealth Apps for CVD Self-Management?

We identified six main functionalities of mHealth apps for CVD self-management (see Table 4):

- Recommendations (F1). Medical recommendations issued for patient follow-up in terms of dietary plans, physical activity, and overall health status.
- Alerts/reminders/text messages (F2). (a) Early, real-time warnings issued before potential vital signal alterations, (b) medication, physical activity, and/or dietary reminders, and (c) text messages communication between patients and physicians.
- Parameter monitoring (F3). Reports of monitored patient parameters, such as active minutes, burned calories, weight, step count, traveled distance, heart rate, blood pressure, body temperature, and physical activity.
- Medication management (F4). Control and follow-up of patient medication.
- Patient medical history (F5). Electronic health records (EHRs) including clinical data, medical history, diagnoses, medications, treatment plans, allergy test records, and laboratory and test results.
- AF detection (F6). Early detection of AF using heart rate monitoring and ECG results.

To summarize our findings on the main functionalities of mHealth apps for CVD self-management, 57.9% of these apps issue medical recommendations to patients [27,28,29,30,31,32,33,34,35,36,37,39,40,45,52,53,54,58,60,61,62,63], whereas 47.3% can generate reminding notifications or alerts for medical appointments [27,29,34,35,36,37,40,45,51,52,54,55,57,58,59,60,62,64]. Additionally, 34.2% of these apps monitor patient parameters, such as physical activity, step count, weight control, blood pressure, heart rate, pulse wave, and body temperature [27,28,29,38,44,48,51,56,57,58,60,64], while 21% allow for AF detection [41,42,43,44,46,47,49,50]. Finally, 21% allow for medication management [27,29,35,37,45,54,59,63,64], and 10.5% allow patients to access their electronic health records [28,45,60,64].

Most of the mHealth applications studied in this work have been demonstrated to be useful in the self-management of CVDs. There is evidence that these applications have changed the behavior of CVDS patients. This can be attributed to the self-alignment of patients to healthier lifestyles and to the constant monitoring of their vital signs. In addition, in the event of any change in patients’ health status, these applications allow relatives and doctors to be notified to provide immediate care and avoid any health complications.

As part of the findings of this research, we identified six main features of the analyzed applications: (1) simplicity of user interface, (2) professional medical assistance, (3) connection with other services, (4) management of medical record, (5) reliable information, and (6) real-time biometric data tracking. In addition, we identified characteristics that are currently not considered in the development of applications to prevent and detect cardiovascular diseases: management of psychological health and family participation. Additionally, we suggest incorporating the following features: virtual rewards/gaming features, social media integration, and data privacy, since they are characteristics commonly sought by users.

The results of the usability tests performed for the mHealth applications have shown that the age factor influences the importance that users give to the applications’ characteristics. Therefore, for children and adolescents, we recommend applications with simple user interfaces, which include social media integration and are oriented towards virtual rewards/gaming. We recommend, however, fully customizable applications with features such as psychological health management and family integration for adult patients.

### 4.4. RQ4. What Are the Major Remarks for Future Work and Challenges to Be Overcome by mHealth Apps for CVD Self-Management?

Since CVD self-management implies dealing with and managing a significant number of data, a lack of comprehensive information may hinder the correct functioning of mHealth apps for CVD self-management. To overcome this problem, many studies recommend implementing scalable app designs and ensuring the interoperability of these apps with other systems. In this sense, we found that only 4 of the 38 applications reviewed allow patients to access their electronic health records, yet this information is crucial both for patients and for CVD self-management.

Additionally, over 60% of the reviewed apps request access to patient personal information without a clear indication of how such information would be stored or used. In this sense, since privacy concerns might affect app usage, application developers should integrate privacy protection measures into their future designs. Other challenges to overcome include improving user satisfaction with respect to app functionalities and supporting patients in their learning of how to use the applications correctly. It is also important that future mHealth apps for CVD self-management address patient psychological health in their design [4]. We also found that none of the reviewed applications possess all the six functionalities for CVD self-management listed in Section 4.3. Hence, we conclude that the apps lack sufficient functions to support patients in effectively self-managing their CVD. Finally, functionalities for patient family involvement have not been sufficiently implemented in these apps.

### 4.5. RQ5. Which Approaches to Data Extraction, Analysis, and Management Are Commonly Implemented in mHealth Apps for CVD Self-Management?

The approaches to data extraction, analysis, and management used by mHealth apps for CVD self-management include machine learning techniques (supervised and unsupervised approaches), machine learning tasks (classification, clustering, regression), big data (structured and unstructured data), and IoT devices/sensors (see Table 5).

Big data make it possible to take advantage of the large amount of information that results from patients accessing health services. These data include, for instance, personal information, electronic medical records, social media data, telehealth data, clinical trials, and even biometric data from wearables [65,66,67]. In this context, we also found that mHealth apps may equally rely on data mining and sentiment analysis techniques. As for association rules and neural networks, they allow mHealth apps to create solutions for better decision making based on real data, thus improving CVD diagnosis, proposing customized treatment plans, reducing medical errors, increasing the effectiveness of CVD prevention measures, and promoting better CVD self-management.

In regard to the IoT devices/sensors, this approach allows mHealth apps to retrieve real-time data on patient biometric variables, such as body temperature, heart rate, and blood pressure, through wearables, which in turn allow physicians to monitor patients remotely [27,28,68,69,70]. Additionally, wearables provide apps with real-time data that facilitate risk factor tracking and prevent CVD events [71]. In this regard, even though IoT platforms can integrate data from medical devices, wearables, and apps, defining data privacy parameters seems to be a considerable challenge to overcome; nevertheless, wearables have been shown to enable effective CVD detection outside of clinics [72].

Finally, mHealth apps for CVD self-management may also resort to machine learning techniques to mainly create predictive models that support—for example—medical diagnosis and treatment plans and predict the evolution of CVDs and their potential complications [27,28,73,74,75,76,77].

### 4.6. RQ6. Which Wearables Are Commonly Used to Detect, Monitor, and/or Identify CVDs?

According to our findings, 85% of the reviewed mHealth apps for CVD self-management rely on smartphones, whereas the remaining 15% use some type of wearable. We identified the five wearable devices most commonly connected to mHealth apps for CVD self-management: chest strap (W1), heart rate monitors (W2), T-shirt-type wearable ECG monitor (W3), the portable ECG monitor (W4), and the smartwatch/smartbands (W5). Table 6 below summarizes such findings. On the other hand, less common devices include pulse oximeters (Sp02 sensors), MiFi devices, and the Hitoe Transmitter 01 device.

We found that it is essential to consider the use of wearables and other types of devices for monitoring biomedical variables automatically. Wearables such as smartwatches and smartbands can successfully assist in CVD detection and prevention. In addition, in most cases, these devices can be synchronized with cloud platforms such as Google Fit, thus storing all the data generated in the cloud. These platforms also allow synchronized data to be retrieved and integrated into mHealth applications.

The Xiaomi Mi Band is one of the most successful families of sport bracelets on the market, whose success could be due to its low price. It works, however, with another mobile application called Mi Fit, which can also be synchronized with Google Fit. We recommend this smartband as a great option for monitoring blood pressure and heart rate with high precision.

### 4.7. RQ7. Which CVD Stages Are Commonly Managed by mHealth Apps?

Many mHealth apps for CVD self-management can support patients throughout multiple stages of a CVD. As can be observed from Table 7, 63.2% of the mHealth apps can manage CVD treatment, 57.9% cover CVD monitoring, 28.9% focus on CVD prevention, and 18.4% allow for CVD diagnosis.

Most of the analyzed applications focused on the treatment of CVDs. These apps were tested by patients diagnosed with a heart disease, showing positive results. We suggest that new mHealth apps focus on the early stages of CVD management, specifically on detection, to allow doctors and patients to prevent medical complications.

## 5. Conclusions

The goal of this scoping review was to describe the current state of mHealth apps for CVD self-management through our analysis of six aspects: (1) CVDs commonly addressed, (2) main functionalities of mHealth apps for CVD self-management, (3) wearables used for CVD detection, monitoring, and identification, (4) disease stages managed by mHealth apps, (5) current approaches to data extraction, analysis, and management, and (6) current challenges to overcome and future work remarks for mHealth apps used in CVD self-management. The scoping review was performed on 38 primary studies, from which we propose the following conclusions: First, arrhythmia is the most common CVD addressed by mHealth apps, with a frequency of 34% (RQ1). Additionally, 63.6% of the mobile applications used by these mHealth apps are compatible with the Android operating system, whereas 13.6% support iOS, and 22.8% support both (RQ2). Additionally, the majority of the reviewed mHealth apps can provide patients medical recommendations, issue medical appointment reminders, and generate notifications for CVD monitoring (RQ3). The two major challenges these applications must overcome are patient resistance to using the technology and the lack of interoperability between mHealth apps and other systems (RQ4). In regard to the approaches for data extraction, analysis, and management, we found that the majority of the mHealth apps for CVD management rely on big data (structured and unstructured data), IoT devices/sensors and machine learning techniques (supervised and unsupervised approaches), and implementing classification, clustering, and regression algorithms (RQ5). Finally, smartphones—specifically Android smartphones—are commonly connected to mHealth apps for CVD self-management, even though wearables are becoming increasingly used (RQ6). Finally, the great majority of mHealth apps for CVD self-management focus on CVD treatment rather than on any other disease phase (RQ7). As regards our suggestions for future work, we first recommend conducting a systematic review of diseases that are correlated with CVD, such as diabetes and hypertension. Likewise, new research efforts should concentrate on exploring the implications of the increasing use of wearables for managing CVDs such as arrhythmia, heart failure, coronary heart disease, and cardiopathies.

## Figures and Tables

**Table 1:** Keywords and related concepts.

Area	Keywords	Related Concepts
Cardiovascular disease	Self-management Self-care Self-monitoring Heart disease Cardiac issues Heart failure Arrhythmia Coronary heart disease Atrial Fibrillation (AF) Hypertension Cardiac arrest Peripheral artery disease	mHealth mobile application smart application wearable smartwatch app

**Table 2:** Comparison of the main characteristics of mHealth apps.

Study Reference	CVD	Main App Functionalities	Challenges and/or Future Work Remarks	Approaches	Device or Web/Mobile Application	CVD Phase
Zisis et al. [27]	Heart failure	Medical recommendations, reminders, weight control	Computer skills of the patient, hearing problems, impaired vision, and cognitive impairment	Supervised machine learning (classification)	Smartphone or Tablet, Heart Failure app	Monitoring, treatment
Bohanec et al. [28]	Heart failure	Nutrition management, managing medication intake, psychological support, daily Exercise management, monitoring biomedical variables, medical recommendations	Increased adaptation to the patients’ lifestyle, add methods for recognizing patients’ activities, and integrating the optimization module in a smart-home environment	Supervised machine learning (random forest algorithm), classic differential evolution algorithm, and IoT device (heart rate, blood pressure)	Wristband, Blood pressure monitor, HeartMan Web app	Monitoring, treatment
Heiney et al. [29]	Heart failure	Text messages for communication between patients and physicians, weight and symptoms control, medical recommendations, medication management	Disparate population with low literacy, low health literacy, and limited smartphone use	IoT device (heart rate)	Smartphone, Healthy Heart app	Monitoring, treatment
Koirala et al. [30]	Heart failure	Medical recommendations	Implement the app in a real environment	Big data type (unstructured data), Supervised machine learning	Smartphone	Prevention, diagnosis
Gonzalez-Sanchez et al. [31]	Heart failure	Medical recommendations	Overcome patient resistance behavior toward using technology Add more functionality to the mobile app	Unsupervised machine learning	Smartphone, Evident II app	Prevention
Barret et al. [32]	Heart failure	Medical recommendations	Measure patient variables Greater focus on CVD asymptomatic patients	Unsupervised machine learning	Smartphone, Abby Web app	Prevention, treatment
Silva et al. [33]	Heart failure	Medical recommendations	Ensure interoperability of mHealth apps for remote monitoring, Heart rate measurement automation	Unsupervised machine learning	Smartphone, MOVIDA.eros app	Monitoring, treatment
Foster [34]	Heart failure	Medical recommendations, alerts	Implement the app in a real environment	Unsupervised machine learning	Smartphone, HF mobile app	Monitoring, treatment
Sakakibara et al. [35]	Heart failure	Medical recommendations, alerts, medication management	Implement the app in a real environment	Big data type (unstructured data)	Smartphone, mobile app	Prevention, treatment
De la Torre-Diez et al. [36]	Heart failure	Medical recommendations, alerts	Integrate the app system with EMR systems, Improve the usability of the mobile app, Add serious games to the app	Unsupervised machine learning	Smartphone, Heartkeeper app	Treatment
K. Rahimi et al. [37]	Heart failure	Medical recommendations, alerts, medication management	Integrate the app system with EMR systems, Increase wearable precision	Unsupervised machine learning, IoT device (heart rate, sensor Sp02)	Smartphone, SUPPORT-HF app, Oximeter	Monitoring, treatment
Bartlett et al. [38]	Heart failure	Step count calculation, weight control, blood pressure control	Overcome technological problems	IoT device (heart rate, blood pressure)	SMART Personalized Self-Management System (PSMS), HTC HD2 phone, MiFi device, mobile app	Monitoring, treatment
Turchioe et al. [39]	Arrhythmia	Medical recommendations	Overcome patient resistance to technology	Unsupervised machine learning	Smartphone	Prevention, monitoring
Pierleoni et al. [40]	Arrhythmia	Medical recommendations, alerts	Implement application in a real environment	Big data type (unstructured data), Unsupervised machine learning	Smartphone	Monitoring, treatment
Reverberi et al. [41]	Arrhythmia	AF detection	Implement algorithm for AF detection	IoT device (heart rate, ECG), Supervised machine learning (classification)	HR monitor of the chest-strap type, RITMIA app	Prevention
Fukuma et al. [42]	Arrhythmia	AF detection	Increase patient monitoring time	IoT device (heart rate, ECG)	T-Shirt-type wearable, ECG monitor, Hitoe Transmitter 01, smartphone	Prevention, treatment
Bumgarner et al. [43]	Arrhythmia	AF detection	Increase sample size, Increase the performance of the KB smartwatch algorithm, Review the real-time display of the ECG recording	IoT device (heart rate, blood pressure), Unsupervised machine learning	Kardia Band, Apple Watch, KB app	Prevention, monitoring
Krivoshei et al. [44]	Arrhythmia	AF detection, monitoring of heart rate, pulse wave analysis	Test the algorithm on a smartwatch	Unsupervised machine learning	Smartphone, iPhone 4S	Prevention
Guo et al. [45]	Arrhythmia	Medical recommendations, medication management, alerts, medical record	Overcome patient resistance to using technology	Supervised machine learning	Smartphone, mAF app	Treatment
Evans et al. [46]	Arrhythmia	AF detection	Extend study to other hospitals serving low-resource areas, Ensure interoperability with further systems	IoT device (heart rate, blood pressure), Supervised machine learning (classification)	AliveCor Kardia mobile ECG device, iPhone and iPad	Diagnosis, monitoring
Halcox et al. [47]	Arrhythmia	AF detection	The relatively high false-positive rate in the minor proportion of those reported as AF by the device	IoT device (heart rate, blood pressure), Supervised machine learning (classification)	AliveCor Kardia device, iPad	Diagnosis, monitoring
Lowres et al. [48]	Arrhythmia	iPhone handheld electrocardiogram (iECG)	Using iECG self-monitoring among other patient groups	Supervised machine learning	iPhone and AliveCor Heart monitor (iECG)	Monitoring
Hickey et al. [49]	Arrhythmia	AF detection	Implement the application in a real environment	IoT device (heart rate, blood pressure), Supervised machine learning (classification)	AliveCor Kardia mobile ECG device, iPhone	Diagnosis, monitoring
McManus et al. [50]	Arrhythmia	AF detection	Improve pulse recording and app performance	IoT device (heart rate), Supervised machine learning (classification)	PULSE-SMART app, iPhone 4S	Diagnosis, monitoring
Kakria et al. [51]	Arrhythmia	Alerts, monitoring of heart rate, blood pressure, and temperature	Solve the problem of delayed alarms in remote areas	IoT device (heart rate, blood pressure, stress level)	Smartphone, Zephyr BT system, G plus sensor, the Omron Wireless Upper Arm blood pressure monitor	Diagnosis, monitoring
Brouwers et al. [52]	Coronary heart disease	Medical recommendations, alerts	Sedentary patients	IoT device (heart rate)	Patient-centered web app, accelerometer, heart rate monitor	Monitoring, treatment
Zhang et al. [53]	Coronary heart disease	Medical recommendations	Ensure interoperability of applications for remote monitoring	Big data type (unstructured data), Unsupervised machine learning	Smartphone, Care4Heart app	Prevention
Athilingam [54]	Coronary heart disease	Medical recommendations, alerts, medication management	Overcome patient resistance to using technology Replace current sensor with handheld sensor	IoT device (heart rate), Supervised machine learning	Smartphone, HeartMapp, BioHarness Bluetooth sensor	Monitoring, treatment
Dale et al. [55]	Coronary heart disease	Text messages for communication of patients and physicians	Implement the app in a real environment	Big data type (structured data)	Smartphone	Treatment
Skobel et al. [56]	Coronary heart disease	Exercise module, activity level monitoring	Automatic arrhythmia detection	IoT device (heart rate, ECG, respiration, activity), Supervised machine learning	HeartCycle’s guided exercise (GEX) system, tablet or laptop, portable PDA for ECG display, shirt with sensors	Diagnosis, monitoring
AM et al. [57]	Coronary heart disease	Educational material, medication reminders, and activity level monitoring	Train medical personnel and patients	IoT device (heart rate)	Smartphone	Monitoring, treatment
Dale et al. [58]	Coronary heart disease	Text messages for communication of patients and physicians, medical recommendations, weight control	Implement app in a real environment	IoT device (heart rate)	Smartphone, web app Text4Heart	Treatment
Jiang et al. [59]	Several (coronary heart disease and hypertension)	Alerts, medication management	Achieve acceptance of mHealth solutions among older patient populations, Improve app design	Supervised machine learning (Regression)	Smartphone, mobile app	Treatment
Baek et al. [60]	Several (atrial fibrillation, hypertension, chest pain, vasovagal syncope, variant angina, and dyspnea on exertion)	Medical recommendations, alerts, diary, weight control	Improve app usability, Integrate app system with EMR (Electronic Medical Record) systems	IoT device (heart rate)	Smartphone	Treatment, monitoring
Supervía & López-Jimenez [61]	Several (heart failure, coronary heart disease, tachycardias, arrhythmia, and hypertension)	Medical recommendations	Guarantee patient data protection and confidentiality	Unsupervised machine learning	Smartphone	Treatment
Tinsel et al. [62]	Several (heart failure, Coronary heart disease, tachycardias, arrhythmia, and hypertension)	Medical recommendations, alerts	Overcome patient resistance to using technology	IoT device (heart rate)	Mobile app	Prevention, treatment
Martorella et al. [63]	Several (heart failure, coronary heart disease, tachycardias, arrhythmia and hypertension)	Medical recommendations, medication management	Screen questionnaire to tailor content according to chronic postsurgical pain (CPSP) risk factors	Not specified	Web app	Monitoring, treatment
Johnston et al. [64]	Several (myocardial infarction, angina pectoris, heart failure, atrial fibrillation, embolic stroke, peripheral artery disease, hypertension)	Medication management, text messaging, reminders, e-diary, exercise module, BMI module, and blood pressure module	Improve patient self-reported drug adherence	IoT device (heart rate)	Smartphone, web-based app	Treatment

**Table 3:** mHealth applications for CVD self-management.

CVD	Study	Mobile App Name	Android	iOS
Heart failure	Zisis et al. [27]	Heart Failure app	✓	
	Bohanec et al. [28]	HeartMan	✓	
	Heiney et al. [29]	Healthy Heart	✓	
	Gonzalez-Sanchez et al. [31]	Evident II	✓	
	Barret et al. [32]	Abby	✓	
	Silva et al. [33]	MOVIDA.eros	✓	✓
	Foster [34]	HF mobile app	✓	✓
	Sakakibara et al. [35] Bartlett et al. [38]	Not specified	✓	
	De la Torre-Diez et al. [36]	HeartKeeper	✓	
	K. Rahimi et al. [37]	SUPPORT-HF	✓	
Arrhythmia	Reverberi et al. [41]	RITMIA	✓	
	Bumgarner et al. [43] Evans et al. [46] Halcox et al. [47] Lowres et al. [48] Hickey et al. [49]	Kardia app		✓
	Krivoshei et al. [44]	Unstated		✓
	Guo et al. [45]	mAF app	✓	✓
	McManus et al. [50]	PULSE-SMART		✓
	Kakria et al. [51]	Not specified	✓	
Coronary heart disease	Zhang et al. [53]	Care4Heart	✓	✓
	Athilingam [54]	HeartMapp	✓	
	AM et al. [57]	Not specified	✓	
	Dale et al. [58]	Text4Heart	✓	
Other CVDs	Jiang et al. [59]	Not specified	✓	
	Supervía & López-Jimenez [61] Tinsel et al. [62]	Not specified	✓	✓

**Table 4:** Main functionalities of mHealth apps for CVD self-management.

CVD	Study	F1	F2	F3	F4	F5	F6
Heart failure	Zisis et al. [27]	✓	✓	✓	✓		
	Bohanec et al. [28]	✓		✓		✓	
	Heiney et al. [29]	✓	✓	✓	✓		
	Koirala et al. [30]	✓					
	Gonzalez-Sanchez et al. [31]	✓					
	Barret et al. [32]	✓					
	Silva et al. [33]	✓					
	Foster [34]	✓	✓				
	Sakakibara et al. [35]	✓	✓		✓		
	De la Torre-Diez et al. [36]	✓	✓				
	K. Rahimi et al. [37]	✓	✓		✓		
	Bartlett et al. [38]			✓			
Arrhythmia	Turchioe et al. [39]	✓					
	Pierleoni et al. [40]	✓	✓				
	Reverberi et al. [41]						✓
	Fukuma et al. [42]						✓
	Bumgarner et al. [43]						✓
	Krivoshei et al. [44]			✓			✓
	Guo et al. [45]	✓	✓		✓	✓	
	Evans et al. [46]						✓
	Halcox et al. [47]						✓
	Lowres et al. [48]			✓			
	Hickey et al. [49]						✓
	McManus et al. [50]						✓
	Kakria et al. [51]		✓	✓			
Coronary heart disease	Brouwers et al. [52]	✓	✓				
	Zhang et al. [53]	✓					
	Athilingam [54]	✓	✓		✓		
	Dale et al. [55]		✓				
	Skobel et al. [56]			✓			
	AM et al. [57]		✓	✓			
	Dale et al. [58]	✓	✓	✓			
Several	Jiang et al. [59]		✓	✓			
	Baek et al. [60]	✓	✓	✓		✓	
	Supervía & López-Jimenez [61]	✓					
	Tinsel et al. [62]	✓	✓				
	Martorella et al. [63]	✓			✓		
	Johnston et al. [64]		✓	✓	✓	✓	

**Table 5:** Main approaches to data extraction and analysis in mHealth apps for CVD self-management.

CVD	Study	Machine Learning Techniques and Tasks	Big Data Types	IoT Devices/Sensors
Heart failure	Zisis et al. [27]	✓		✓
	Bohanec et al. [28]	✓		✓
	Heiney et al. [29]			✓
	Koirala et al. [30]	✓	✓	
	Gonzalez-Sanchez et al. [31]	✓		
	Barret et al. [32]	✓		
	Silva et al. [33]	✓		
	Foster [34]	✓		
	Sakakibara et al. [35]		✓	
	De la Torre-Diez et al. [36]	✓		
	K. Rahimi et al. [37]	✓		✓
	Bartlett et al. [38]			✓
Arrhythmia	Turchioe et al. [39]	✓		
	Pierleoni et al. [40]	✓	✓	
	Reverberi et al. [41]	✓		
	Fukuma et al. [42]			✓
	Bumgarner et al. [43]	✓		✓
	Krivoshei et al. [44]	✓		
	Guo et al. [45]	✓		
	Evans et al. [46]	✓		✓
	Halcox et al. [47]	✓		✓
	Lowres et al. [48]			✓
	Hickey et al. [49]	✓		✓
	McManus et al. [50]	✓		✓
	Kakria et al. [51]			✓
Coronary heart disease	Brouwers et al. [52]			✓
	Zhang et al. [53]	✓	✓	
	Athilingam [54]	✓		
	Skobel et al. [56]	✓		✓
	AM et al. [57]			✓
	Dale et al. [58]			✓
Several	Jiang et al. [59]	✓		
	Baek et al. [60]			✓
	Supervía & López-Jimenez [61]	✓		
	Tinsel et al. [62]			✓

**Table 6:** Main Wearables for CVD Monitoring.

CVD	Study	W1	W2	W3	W4	W5
Heart failure	Bohanec et al. [28]		✓			✓
	Bartlett et al. [38]		✓			
Arrhythmia	Reverberi et al. [41]		✓			
	Fukuma et al. [42]			✓		
	Bumgarner et al. [43]				✓	✓
	Evans et al. [46]				✓	✓
	Halcox et al. [47]				✓	✓
	Lowres et al. [48]				✓	✓
	Hickey et al. [49]				✓	✓
	Kakria et al. [51]	✓	✓			
Coronary heart disease	Brouwers et al. [52]		✓			
	Athilingam [54]	✓	✓			
	Skobel et al. [56]			✓	✓	

**Table 7:** Disease stages managed by mHealth apps for CVD self-management.

CVD	Study	Prevention	Diagnosis	Monitoring	Treatment
Heart failure	Zisis et al. [27]			✓	✓
	Bohanec et al. [28]			✓	✓
	Heiney et al. [29]			✓	✓
	Koirala et al. [30]	✓	✓		
	Gonzalez-Sanchez et al. [31]	✓			
	Barret et al. [32]	✓			✓
	Silva et al. [33]			✓	✓
	Foster [34]			✓	✓
	Sakakibara et al. [35]	✓			✓
	De la Torre-Diez et al. [36]				✓
	K. Rahimi et al. [37]			✓	✓
	Bartlett et al. [38]			✓	✓
Arrhythmia	Turchioe et al. [39]	✓		✓	
	Pierleoni et al. [40]			✓	✓
	Reverberi et al. [41]	✓			
	Fukuma et al. [42]	✓			✓
	Bumgarner et al. [43]	✓		✓	
	Krivoshei et al. [44]	✓			
	Guo et al. [45]				✓
	Evans et al. [46]		✓	✓	
	Halcox et al. [47]		✓	✓	
	Lowres et al. [48]			✓	
	Hickey et al. [49]		✓	✓	
	McManus et al. [50]		✓	✓	
	Kakria et al. [51]		✓	✓	
Coronary heart disease	Brouwers et al. [52]			✓	✓
	Zhang et al. [53]	✓			
	Athilingam [54]			✓	✓
	Dale et al. [55]				✓
	Skobel et al. [56]		✓	✓	
	AM et al. [57]			✓	✓
	Dale et al. [58]				✓
Several	Jiang et al. [59]				✓
	Baek et al. [60]			✓	✓
	Supervía & López-Jimenez [61]				✓
	Tinsel et al. [62]	✓			✓
	Martorella et al. [63]			✓	✓
	Johnston et al. [64]				✓

## Data Availability

Not applicable.

## References

[1] WHO, “Noncommunicable Diseases”, World Health Organization (WHO), 13 April 2017. https://www.who.int/en/news-room/fact-sheets/detail/noncommunicable-diseases.

[2] Athilingam P., Jenkins B. (2018). Mobile Phone Apps to Support Heart Failure Self-Care Management: Integrative Review. JMIR Cardio.

[3] Chow C.K., Ariyarathna N., Islam S.M.S., Thiagalingam A., Redfern J. (2016). mHealth in Cardiovascular Health Care. Heart Lung Circ..

[4] Xie B., Su Z., Zhang W., Cai R. (2017). Chinese Cardiovascular Disease Mobile Apps’ Information Types, Information Quality, and Interactive Functions for Self-Management: Systematic Review. JMIR mHealth uHealth.

[5] Searcy R.P., Summapund J., Estrin D., Pollak J.P., Schoenthaler A., Troxel A.B., Dodson J.A. (2019). Mobile Health Technologies for Older Adults with Cardiovascular Disease: Current Evidence and Future Directions. Curr. Geriatr. Rep..

[6] Coorey G.M., Neubeck L., Mulley J., Redfern J. (2018). Effectiveness, acceptability and usefulness of mobile applications for cardiovascular disease self-management: Systematic review with meta-synthesis of quantitative and qualitative data. Eur. J. Prev. Cardiol..

[7] Dale L.P., Dobson R., Whittaker R., Maddison R. (2016). The effectiveness of mobile-health behaviour change interventions for cardiovascular disease self-management: A systematic review. Eur. J. Prev. Cardiol..

[8] Whitehead L., Seaton P. (2016). The Effectiveness of Self-Management Mobile Phone and Tablet Apps in Long-term Condition Management: A Systematic Review. J. Med. Internet Res..

[9] Gandhi S., Chen S., Hong L., Sun K., Gong E., Li C., Yan L.L., Schwalm J.-D. (2017). Effect of Mobile Health Interventions on the Secondary Prevention of Cardiovascular Disease: Systematic Review and Meta-analysis. Can. J. Cardiol..

[10] Pearsons A., Hanson C.L., Gallagher R., O’Carroll R.E., Khonsari S., Hanley J., Strachan F.E., Mills N.L., Quinn T.J., McKinstry B. (2021). Atrial fibrillation self-management: A mobile telephone app scoping review and content analysis. Eur. J. Cardiovasc. Nurs..

[11] Villarreal V., Alvarez A. (2020). Evaluation of mHealth Applications Related to Cardiovascular Diseases: A Systematic Review. Acta Inform. Med..

[12] Neubeck L., Lowres N., Benjamin E., Freedman B., Coorey G., Redfern J. (2015). The mobile revolution—using smartphone apps to prevent cardiovascular disease. Nat. Rev. Cardiol..

[13] Cruz-Martínez R.R., Wentzel J., Asbjørnsen R.A., Noort P.D., van Niekerk J.M., Sanderman R., van Gemert-Pijnen J.E. (2020). Supporting Self-Management of Cardiovascular Diseases Through Remote Monitoring Technologies: Metaethnography Review of Frameworks, Models, and Theories Used in Research and Development. J. Med. Internet Res..

[14] Hannan A.L., Harders M.P., Hing W., Climstein M., Coombes J.S., Furness J. (2019). Impact of wearable physical activity monitoring devices with exercise prescription or advice in the maintenance phase of cardiac rehabilitation: Systematic review and meta-analysis. BMC Sports Sci. Med. Rehabil..

[15] Marston H.R., Hadley R., Banks D., Duro M.D.C.M. (2019). Mobile Self-Monitoring ECG Devices to Diagnose Arrhythmia that Coincide with Palpitations: A Scoping Review. Healthcare.

[16] Brørs G., Pettersen T.R., Hansen T.B., Fridlund B., Hølvold L.B., Lund H., Norekvål T.M. (2019). Modes of e-Health delivery in secondary prevention programmes for patients with coronary artery disease: A systematic review. BMC Health Serv. Res..

[17] Villarreal V., Castillo-Sanchez G., Hamrioui S., Alvarez A.B., Díez I.D.L.T., Lorenz P. (2018). A Systematic Review of mHealth apps Evaluations for Cardiac Issues. Multidiscip. Digit. Publ. Inst. Proc..

[18] Hamilton S.J., Mills B., Birch E.M., Thompson S.C. (2018). Smartphones in the secondary prevention of cardiovascular disease: A systematic review. BMC Cardiovasc. Disord..

[19] Bochicchio M.A., Vaira L., Mortara A., De Maria R. A preliminar analysis and comparison of international projects on mobile devices and mHealth Apps for heart failure. Proceedings of the 2019 5th Experiment International Conference (exp.at’19).

[20] Allida S., Du H., Xu X., Prichard R., Chang S., Hickman L.D., Davidson P.M., Inglis S.C. (2020). mHealth education interventions in heart failure. Cochrane Database Syst. Rev..

[21] Schmaderer M.S., Struwe L., Loecker C., Lier L., Lundgren S.W., Wichman C., Pozehl B., Zimmerman L. (2021). Mobile Health Self-management Interventions for Patients With Heart Failure. J. Cardiovasc. Nurs..

[22] Creber R.M.M., Maurer M.S., Reading M., Hiraldo G., Hickey K.T., Iribarren S. (2016). Review and Analysis of Existing Mobile Phone Apps to Support Heart Failure Symptom Monitoring and Self-Care Management Using the Mobile Application Rating Scale (MARS). JMIR mHealth uHealth.

[23] Arksey H., O’Malley L. (2005). Scoping studies: Towards a methodological framework. Int. J. Soc. Res. Methodol..

[24] Levac D., Colquhoun H., O’Brien K.K. (2010). Scoping studies: Advancing the methodology. Implement. Sci..

[25] Moher D., Liberati A., Tetzlaff J., Altman D.G., PRISMA Group (2009). Preferred reporting items for systematic reviews and meta-analyses: The PRISMA statement. PLoS Med..

[26] Tricco A.C., Lillie E., Zarin W., O’Brien K.K., Colquhoun H., Levac D., Moher D., Peters M.D.J., Horsley T., Weeks L. (2018). PRISMA extension for scoping reviews (PRISMA-ScR): Checklist and explanation. Ann. Intern. Med..

[27] Zisis G., Carrington M.J., Oldenburg B., Whitmore K., Lay M., Huynh Q., Neil C., Ball J., Marwick T.H. (2021). An m-Health intervention to improve education, self-management, and outcomes in patients admitted for acute decompensated heart failure: Barriers to effective implementation. Eur. Heart J. Digit. Health.

[28] Bohanec M., Tartarisco G., Marino F., Pioggia G., Puddu P.E., Schiariti M.S., Baert A., Pardaens S., Clays E., Vodopija A. (2021). HeartMan DSS: A decision support system for self-management of congestive heart failure. Expert Syst. Appl..

[29] Heiney S.P., Donevant S.B., Adams S.A., Parker P.D., Chen H., Levkoff S. (2020). A Smartphone App for Self-Management of Heart Failure in Older African Americans: Feasibility and Usability Study. JMIR Aging.

[30] Koirala B., Himmelfarb C.R.D., Budhathoki C., Davidson P.M. (2020). Heart failure self-care, factors influencing self-care and the relationship with health-related quality of life: A cross-sectional observational study. Heliyon.

[31] Gonzalez-Sanchez J., Recio-Rodriguez J.I., Fernandez-Delrio A., Sanchez-Perez A., Magdalena-Belio J.F., Gomez-Marcos M.A., Garcia-Ortiz L. (2019). Using a smartphone app in changing cardiovascular risk factors: A randomized controlled trial (EVIDENT II study). Int. J. Med. Inform..

[32] Barrett M., Boyne J., Brandts J., Rocca H.-P.B.-L., De Maesschalck L., De Wit K., Dixon L., Eurlings C., Fitzsimons D., Golubnitschaja O. (2019). Artificial intelligence supported patient self-care in chronic heart failure: A paradigm shift from reactive to predictive, preventive and personalised care. EPMA J..

[33] Silva E., Rijo R., Martinho R., Assuncao P., Seco A., Fonseca-Pinto R. (2018). A Cardiac Rehabilitation Program Supported by mHealth Technology: The MOVIDA.eros Platform. Procedia Comput. Sci..

[34] Foster M. (2018). A Mobile Application for Patients with Heart Failure: Theory—and Evidence-Based Design and Testing. CIN Comput. Inform. Nurs..

[35] Sakakibara B.M., Ross E., Arthur G., Brown-Ganzert L., Petrin S., Sedlak T., Lear S.A. (2017). Using Mobile-Health to Connect Women with Cardiovascular Disease and Improve Self-Management. Telemed. e-Health.

[36] De la Torre-Diez I., Martinez-Perez B., Lopez-Coronado M., Rodrigues J.J.P.C., Arambarri J. Development and validation of a mobile health app for the self-management and education of cardiac patients. Proceedings of the 2016 11th Iberian Conference on Information Systems and Technologies (CISTI).

[37] Rahimi K., Velardo C., Triantafyllidis A., Conrad N., Shah S.A., Chantler T., Mohseni H., Stoppani E., Moore F., Paton C. (2015). A user-centred home monitoring and self-management system for patients with heart failure: A multicentre cohort study. Eur. Heart J. Qual. Care Clin. Outcomes.

[38] Bartlett Y.K., Haywood A., Bentley C.L., Parker J., Hawley M.S., Mountain G.A., Mawson S. (2014). The SMART personalised self-management system for congestive heart failure: Results of a realist evaluation. BMC Med. Inform. Decis. Mak..

[39] Turchioe M.R., Jimenez V., Isaac S., Alshalabi M., Slotwiner D., Creber R.M. (2020). Review of mobile applications for the detection and management of atrial fibrillation. Heart Rhythm O2.

[40] Pierleoni P., Belli A., Gentili A., Incipini L., Palma L., Raggiunto S., Sbrollini A., Burattini L. (2021). Real-time smart monitoring system for atrial fibrillation pathology. J. Ambient Intell. Humaniz. Comput..

[41] Reverberi C., Rabia G., De Rosa F., Bosi D., Botti A., Benatti G. (2019). The RITMIATM Smartphone App for Automated Detection of Atrial Fibrillation: Accuracy in Consecutive Patients Undergoing Elective Electrical Cardioversion. BioMed Res. Int..

[42] Fukuma N., Hasumi E., Fujiu K., Waki K., Toyooka T., Komuro I., Ohe K. (2019). Feasibility of a T-Shirt-Type Wearable Electrocardiography Monitor for Detection of Covert Atrial Fibrillation in Young Healthy Adults. Sci. Rep..

[43] Bumgarner J.M., Lambert C.T., Hussein A.A., Cantillon D.J., Baranowski B., Wolski K., Lindsay B.D., Wazni O.M., Tarakji K.G. (2018). Smartwatch Algorithm for Automated Detection of Atrial Fibrillation. J. Am. Coll. Cardiol..

[44] Krivoshei L., Weber S., Burkard T., Maseli A., Brasier N., Kühne M., Conen D., Huebner T., Seeck A., Eckstein J. (2016). Smart detection of atrial fibrillation. Europace.

[45] Guo Y., Chen Y., Lane D.A., Liu L., Wang Y., Lip G.Y. (2017). Mobile Health Technology for Atrial Fibrillation Management Integrating Decision Support, Education, and Patient Involvement: mAF App Trial. Am. J. Med..

[46] Evans G.F., Shirk A., Muturi P., Soliman E.Z. (2017). Feasibility of Using Mobile ECG Recording Technology to Detect Atrial Fibrillation in Low-Resource Settings. Glob. Heart.

[47] Halcox J.P., Wareham K., Cardew A., Gilmore M., Barry J.P., Phillips C., Gravenor M.B. (2017). Assessment of remote heart rhythm sampling using the AliveCor heart monitor to screen for atrial fibrillation the REHEARSE-AF study. Circulation.

[48] Lowres N., Mulcahy G., Gallagher R., Ben Freedman S., Marshman D., Kirkness A., Orchard J., Neubeck L. (2016). Self-monitoring for atrial fibrillation recurrence in the discharge period post-cardiac surgery using an iPhone electrocardiogram. Eur. J. Cardio-Thorac. Surg..

[49] Hickey K.T., Hauser N.R., Valente L.E., Riga T.C., Frulla A.P., Creber R.M., Whang W., Garan H., Jia H., Sciacca R.R. (2016). A single-center randomized, controlled trial investigating the efficacy of a mHealth ECG technology intervention to improve the detection of atrial fibrillation: The iHEART study protocol. BMC Cardiovasc. Disord..

[50] McManus D.D., Chong J.W., Soni A., Saczynski J.S., Esa N., Napolitano C., Darling C.E., Boyer E., Rosen R.K., Floyd K.C. (2015). PULSE-SMART: Pulse-Based Arrhythmia Discrimination Using a Novel Smartphone Application. J. Cardiovasc. Electrophysiol..

[51] Kakria P., Tripathi N.K., Kitipawang P. (2015). A Real-Time Health Monitoring System for Remote Cardiac Patients Using Smartphone and Wearable Sensors. Int. J. Telemed. Appl..

[52] Brouwers R.W., Kraal J.J., Traa S.C., Spee R.F., Oostveen L.M., Kemps H.M. (2017). Effects of cardiac telerehabilitation in patients with coronary artery disease using a personalised patient-centred web application: Protocol for the SmartCare-CAD randomised controlled trial. BMC Cardiovasc. Disord..

[53] Zhang H., Jiang Y., Nguyen H.D., Poo D.C.C., Wang W. (2017). The effect of a smartphone-based coronary heart disease prevention (SBCHDP) programme on awareness and knowledge of CHD, stress, and cardiac-related lifestyle behaviours among the working population in Singapore: A pilot randomised controlled trial. Health Qual. Life Outcomes.

[54] Athilingam P., Labrador M.A., Remo E.F.J., Mack L., San Juan A.B., Elliott A.F. (2016). Features and usability assessment of a patient-centered mobile application (HeartMapp) for self-management of heart failure. Appl. Nurs. Res..

[55] Dale L.P., Whittaker R., Jiang Y., Stewart R., Rolleston A., Maddison R. (2015). Text Message and Internet Support for Coronary Heart Disease Self-Management: Results From the Text4Heart Randomized Controlled Trial. J. Med. Internet Res..

[56] Skobel E., Martinez-Romero A., Scheibe B., Schauerte P., Marx N., Luprano J., Knackstedt C. (2014). Evaluation of a newly designed shirt-based ECG and breathing sensor for home-based training as part of cardiac rehabilitation for coronary artery disease. Eur. J. Prev. Cardiol..

[57] Layton A.M., Whitworth J., Peacock J., Bartels M.N., Jellen P.A., Thomashow B.M. (2014). Feasibility and Acceptability of Utilizing a Smartphone Based Application to Monitor Outpatient Discharge Instruction Compliance in Cardiac Disease Patients around Discharge from Hospitalization. Int. J. Telemed. Appl..

[58] Dale L.P., Whittaker R., Jiang Y., Stewart R., Rolleston A., Maddison R. (2014). Improving coronary heart disease self-management using mobile technologies (Text4Heart): A randomised controlled trial protocol. Trials.

[59] Jiang J., Zhu Q., Zheng Y., Zhu Y., Li Y., Huo Y. (2019). Perceptions and Acceptance of mHealth in Patients With Cardiovascular Diseases: A Cross-Sectional Study. JMIR mHealth uHealth.

[60] Baek H., Suh J.-W., Kang S.-H., Kang S., Lim T.H., Hwang H., Yoo S. (2018). Enhancing User Experience Through User Study: Design of an mHealth Tool for Self-Management and Care Engagement of Cardiovascular Disease Patients. JMIR Cardio.

[61] Supervía M., López-Jimenez F. (2018). mHealth and cardiovascular diseases self-management: There is still a long way ahead of us. Eur. J. Prev. Cardiol..

[62] Tinsel I., Siegel A., Schmoor C., Poguntke I., Maun A., Niebling W. (2018). Encouraging Self-Management in Cardiovascular Disease Prevention. Dtsch. Ärztebl. Int..

[63] Martorella G., Graven L., Schluck G., Bérubé M., Gélinas C. (2018). Nurses’ Perception of a Tailored Web-Based Intervention for the Self-Management of Pain After Cardiac Surgery. SAGE Open Nurs..

[64] Johnston N., Bodegard J., Jerström S., Åkesson J., Brorsson H., Alfredsson J., Albertsson P.A., Karlsson J.-E., Varenhorst C. (2016). Effects of interactive patient smartphone support app on drug adherence and lifestyle changes in myocardial infarction patients: A randomized study. Am. Heart J..

[65] Gökalp M.O., Kayabay K., Akyol M.A., Koçyiğit A., Eren P.E. (2018). Big data in mHealth. Current and Emerging mHealth Technologies: Adoption, Implementation, and Use.

[66] Khan Z.F., Alotaibi S.R. (2020). Applications of Artificial Intelligence and Big Data Analytics in m-Health: A Healthcare System Perspective. J. Healthc. Eng..

[67] Baladrón C., de Diego J.J.G., Amat-Santos I.J. (2021). Big data and new information technology: What cardiologists need to know. Rev. Esp. Cardiol..

[68] Code R. (2019). Wearable technology in healthcare. Nat. Biotechnol..

[69] Singhal A., Cowie M.R. (2020). The Role of Wearables in Heart Failure. Curr. Heart Fail. Rep..

[70] Dagher L., Shi H., Zhao Y., Marrouche N.F. (2020). Wearables in cardiology: Here to stay. Heart Rhythm.

[71] Kario K. (2020). Management of Hypertension in the Digital Era: Small Wearable Monitoring Devices for Remote Blood Pressure Monitoring. Hypertension.

[72] Dunn J., Runge R., Snyder M. (2018). Wearables and the medical revolution. Pers. Med..

[73] Ambhore S. Early Detection of Cardiovascular Diseases Using Deep Convolutional Neural Network & Fourier Wavelet Transform. https://www.sciencedirect.com/science/article/pii/S2214785320392324.

[74] Mohan S., Thirumalai C., Srivastava G. (2019). Effective Heart Disease Prediction Using Hybrid Machine Learning Techniques. IEEE Access.

[75] Khan M.A. (2020). An IoT Framework for Heart Disease Prediction Based on MDCNN Classifier. IEEE Access.

[76] Raj S. (2020). An Efficient IoT-Based Platform for Remote Real-Time Cardiac Activity Monitoring. IEEE Trans. Consum. Electron..

[77] Khan M.A., Algarni F. (2020). A Healthcare Monitoring System for the Diagnosis of Heart Disease in the IoMT Cloud Environment Using MSSO-ANFIS. IEEE Access.

